# Bis(4-acetyl­anilinium) hexa­chlorido­stannate(IV)

**DOI:** 10.1107/S1600536811015546

**Published:** 2011-04-29

**Authors:** Xian-Wang Song, Rui-Ting Xue, Shou-Gang Chen, Yan-Sheng Yin

**Affiliations:** a Institute of Materials Science and Engineering, Ocean University of China, Qingdao, Shandong 266100, People’s Republic of China

## Abstract

In the title compound, (C_8_H_10_NO)_2_[SnCl_6_], the Sn^IV^ atom exists in an octa­hedral coordination environment. In the crystal, inter­molecular N—H⋯O and N—H⋯Cl hydrogen bonds link the cations and anions into a three-dimensional framework.

## Related literature

For general background to inorganic–organic hybrid compounds, see: Antonietti & Ozin (2004 ▶); Cong & Yu (2009 ▶); Descazo *et al.* (2006 ▶); Li *et al.* (2007 ▶); Sanchez *et al.* (2005 ▶).
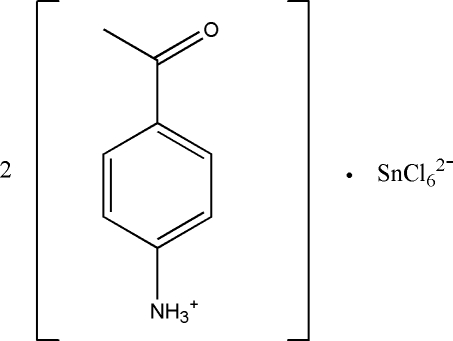


## Experimental

### 

#### Crystal data

(C_8_H_10_NO)_2_[SnCl_6_]
*M* *_r_* = 603.73
Monoclinic, 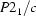

*a* = 7.2540 (8) Å
*b* = 12.6481 (13) Å
*c* = 24.438 (2) Å
β = 93.991 (1)°
*V* = 2236.7 (4) Å^3^
*Z* = 4
Mo *K*α radiation
μ = 1.87 mm^−1^
*T* = 298 K
0.48 × 0.44 × 0.43 mm

#### Data collection

Bruker SMART CCD area-detector diffractometer
Absorption correction: multi-scan (*SADABS*; Sheldrick, 1996 ▶) *T*_min_ = 0.467, *T*_max_ = 0.500
10422 measured reflections
3929 independent reflections
2996 reflections with *I* > 2σ(*I*)
*R*_int_ = 0.058

#### Refinement

*R*[*F* ^2^ > 2σ(*F* ^2^)] = 0.044
*wR*(*F* ^2^) = 0.112
*S* = 1.00
3929 reflections
248 parameters
H-atom parameters constrained
Δρ_max_ = 0.84 e Å^−3^
Δρ_min_ = −0.83 e Å^−3^

### 

Data collection: *SMART* (Bruker, 1997 ▶); cell refinement: *SAINT* (Bruker, 1997 ▶); data reduction: *SAINT*; program(s) used to solve structure: *SHELXS97* (Sheldrick, 2008 ▶); program(s) used to refine structure: *SHELXL97* (Sheldrick, 2008 ▶); molecular graphics: *XP* in *SHELXTL* (Sheldrick, 2008 ▶); software used to prepare material for publication: *SHELXL97*.

## Supplementary Material

Crystal structure: contains datablocks global, I. DOI: 10.1107/S1600536811015546/ci5184sup1.cif

Structure factors: contains datablocks I. DOI: 10.1107/S1600536811015546/ci5184Isup2.hkl

Additional supplementary materials:  crystallographic information; 3D view; checkCIF report

## Figures and Tables

**Table 1:** Hydrogen-bond geometry (Å, °)

*D*—H⋯*A*	*D*—H	H⋯*A*	*D*⋯*A*	*D*—H⋯*A*
N2—H2*A*⋯O1^i^	0.89	2.06	2.939 (7)	170
N1—H1*B*⋯O2^ii^	0.89	2.01	2.884 (6)	168
N1—H1*A*⋯Cl1^iii^	0.89	2.49	3.322 (6)	156
N2—H2*B*⋯Cl2^i^	0.89	2.59	3.350 (6)	144
N2—H2*C*⋯Cl3^iv^	0.89	2.69	3.321 (5)	129
N1—H1*C*⋯Cl5^v^	0.89	2.55	3.367 (5)	153
N2—H2*C*⋯Cl6^iv^	0.89	2.64	3.442 (5)	151

Symmetry codes: (i) 
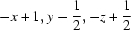
; (ii) 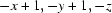; (iii) 
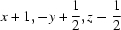
; (iv) 
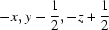
; (v) 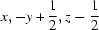.

**Fig. 1.**
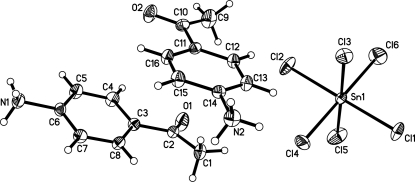
The structure of the title compound, showing 30% probability displacement ellipsoids and the atom-numbering scheme.

## supplementary crystallographic information

### Comment

Inorganic-organic hybrid materials have received much attention due to their potential applications in many areas such as gas storage, separation, catalysis, magnetism, optics as well as in electrical conductivity (Descazo *et al.*, 2006; Li *et al.*, 2007; Sanchez *et al.*, 2005). Recently, we have prepared the title compound and here its crystal structure is reported.

This title compound contains SnC1_6_ inorganic anions and organic cations. The SnCl_6_ inorganic anion adopts a regular octahedral geometry, with average Sn—Cl distance of 2.4102 Å. In the organic cations, the diangle between the methyl ketone and the phenyl ring is 14.9 (3)° or 3.1 (2)°.

In the crystal structure, intermolecular N—H···O and N—H···Cl hydrogen bonds link cations and anions into a three-dimensioal framework.

### Experimental

p-Aminoacetophenone (10 mmol) was dissolved in methanol (10 ml). Ten minutes later, a methanol solution (10 ml) of tin tetrachloride (5 mmol) was added with stirring. The reaction mixture was stirred for 4 h. The solution was held at room temperature for about two weeks, whereupon yellow crystals suitable for X-ray diffraction analysis were obtained.

### Refinement

All H-atoms were positioned geometrically and refined using a riding model, with C–H = 0.96 Å (methyl), 0.93 Å (aromatic), N–H = 0.89 Å (ammonium) and *U*_iso_(H) = 1.5*U*_eq_(C_methyl_,N) and 1.2*U*_eq_(C).

### Crystal data

**Table:**

(C_8_H_10_NO)_2_[SnCl_6_]	*F*(000) = 1192
*M**_r_* = 603.73	*D*_x_ = 1.793 Mg m^−^^3^
Monoclinic, *P*2_1_/*c*	Mo *K*α radiation, λ = 0.71073 Å
Hall symbol: -P 2ybc	Cell parameters from 3529 reflections
*a* = 7.2540 (8) Å	θ = 2.5–27.3°
*b* = 12.6481 (13) Å	µ = 1.87 mm^−^^1^
*c* = 24.438 (2) Å	*T* = 298 K
β = 93.991 (1)°	Block, yellow
*V* = 2236.7 (4) Å^3^	0.48 × 0.44 × 0.43 mm
*Z* = 4	

### Data collection

**Table:**

Bruker SMART CCD area-detector diffractometer	3929 independent reflections
Radiation source: fine-focus sealed tube	2996 reflections with *I* > 2σ(*I*)
graphite	*R*_int_ = 0.058
φ and ω scans	θ_max_ = 25.0°, θ_min_ = 1.7°
Absorption correction: multi-scan (*SADABS*; Sheldrick, 1996)	*h* = −8→8
*T*_min_ = 0.467, *T*_max_ = 0.500	*k* = −15→14
10422 measured reflections	*l* = −23→29

### Refinement

**Table:**

Refinement on *F*^2^	Primary atom site location: structure-invariant direct methods
Least-squares matrix: full	Secondary atom site location: difference Fourier map
*R*[*F*^2^ > 2σ(*F*^2^)] = 0.044	Hydrogen site location: inferred from neighbouring sites
*wR*(*F*^2^) = 0.112	H-atom parameters constrained
*S* = 1.00	*w* = 1/[σ^2^(*F*_o_^2^) + (0.0414*P*)^2^ + 4.9089*P*] where *P* = (*F*_o_^2^ + 2*F*_c_^2^)/3
3929 reflections	(Δ/σ)_max_ = 0.001
248 parameters	Δρ_max_ = 0.84 e Å^−^^3^
0 restraints	Δρ_min_ = −0.83 e Å^−^^3^

### Special details

**Table:**

Geometry. All e.s.d.'s (except the e.s.d. in the dihedral angle between two l.s. planes) are estimated using the full covariance matrix. The cell e.s.d.'s are taken into account individually in the estimation of e.s.d.'s in distances, angles and torsion angles; correlations between e.s.d.'s in cell parameters are only used when they are defined by crystal symmetry. An approximate (isotropic) treatment of cell e.s.d.'s is used for estimating e.s.d.'s involving l.s. planes.
Refinement. Refinement of *F*^2^ against ALL reflections. The weighted *R*-factor *wR* and goodness of fit *S* are based on *F*^2^, conventional *R*-factors *R* are based on *F*, with *F* set to zero for negative *F*^2^. The threshold expression of *F*^2^ > σ(*F*^2^) is used only for calculating *R*-factors(gt) *etc*. and is not relevant to the choice of reflections for refinement. *R*-factors based on *F*^2^ are statistically about twice as large as those based on *F*, and *R*- factors based on ALL data will be even larger.

### Fractional atomic coordinates and isotropic or equivalent isotropic displacement parameters (Å^2^)

**Table:**

	*x*	*y*	*z*	*U*_iso_*/*U*_eq_	
Sn1	0.26788 (5)	0.33553 (3)	0.392680 (15)	0.02996 (14)	
Cl1	0.2381 (3)	0.19836 (12)	0.46029 (6)	0.0541 (5)	
Cl2	0.2941 (3)	0.47370 (13)	0.32429 (7)	0.0631 (5)	
Cl3	0.0010 (2)	0.26670 (12)	0.33926 (7)	0.0492 (4)	
Cl4	0.4656 (3)	0.22042 (16)	0.34441 (7)	0.0645 (5)	
Cl5	0.5344 (3)	0.40331 (15)	0.44385 (9)	0.0763 (6)	
Cl6	0.0643 (3)	0.44692 (15)	0.43985 (8)	0.0706 (6)	
N1	0.7808 (7)	0.3221 (4)	−0.04410 (19)	0.0425 (12)	
H1A	0.8949	0.3038	−0.0516	0.064*	
H1B	0.7532	0.3847	−0.0591	0.064*	
H1C	0.7011	0.2739	−0.0578	0.064*	
N2	0.2441 (7)	−0.0116 (4)	0.1716 (2)	0.0435 (13)	
H2A	0.2336	−0.0262	0.2069	0.065*	
H2B	0.3501	−0.0378	0.1612	0.065*	
H2C	0.1501	−0.0406	0.1515	0.065*	
O1	0.7432 (7)	0.4341 (3)	0.21126 (18)	0.0580 (13)	
O2	0.2530 (7)	0.4680 (3)	0.08980 (18)	0.0539 (12)	
C1	0.7034 (9)	0.2499 (5)	0.2194 (2)	0.0438 (15)	
H1D	0.6742	0.2672	0.2560	0.066*	
H1E	0.8139	0.2079	0.2207	0.066*	
H1F	0.6032	0.2106	0.2015	0.066*	
C2	0.7326 (8)	0.3483 (5)	0.1884 (2)	0.0367 (14)	
C3	0.7471 (8)	0.3413 (4)	0.1275 (2)	0.0311 (12)	
C4	0.7306 (9)	0.4318 (4)	0.0953 (2)	0.0418 (15)	
H4	0.7124	0.4969	0.1117	0.050*	
C5	0.7409 (9)	0.4261 (4)	0.0395 (2)	0.0409 (15)	
H5	0.7288	0.4865	0.0179	0.049*	
C6	0.7697 (8)	0.3287 (4)	0.0163 (2)	0.0323 (13)	
C7	0.7890 (8)	0.2386 (4)	0.0465 (2)	0.0357 (14)	
H7	0.8114	0.1742	0.0298	0.043*	
C8	0.7747 (8)	0.2445 (4)	0.1024 (2)	0.0352 (13)	
H8	0.7836	0.1833	0.1235	0.042*	
C9	0.2098 (11)	0.5103 (5)	0.1816 (3)	0.060 (2)	
H9A	0.2172	0.5817	0.1685	0.091*	
H9B	0.3052	0.4986	0.2102	0.091*	
H9C	0.0912	0.4990	0.1957	0.091*	
C10	0.2339 (8)	0.4365 (4)	0.1361 (3)	0.0367 (14)	
C11	0.2402 (7)	0.3190 (4)	0.1471 (2)	0.0291 (12)	
C12	0.2097 (8)	0.2773 (4)	0.1983 (2)	0.0334 (13)	
H12	0.1874	0.3226	0.2271	0.040*	
C13	0.2122 (8)	0.1688 (4)	0.2070 (2)	0.0361 (13)	
H13	0.1942	0.1407	0.2414	0.043*	
C14	0.2417 (7)	0.1046 (4)	0.1635 (2)	0.0309 (13)	
C15	0.2731 (9)	0.1431 (4)	0.1123 (2)	0.0393 (15)	
H15	0.2941	0.0973	0.0836	0.047*	
C16	0.2725 (8)	0.2515 (4)	0.1044 (2)	0.0368 (14)	
H16	0.2939	0.2790	0.0701	0.044*	

### Atomic displacement parameters (Å^2^)

**Table:**

	*U*^11^	*U*^22^	*U*^33^	*U*^12^	*U*^13^	*U*^23^
Sn1	0.0358 (2)	0.0256 (2)	0.0283 (2)	−0.00138 (17)	0.00080 (15)	0.00247 (16)
Cl1	0.0862 (13)	0.0402 (9)	0.0365 (9)	−0.0050 (8)	0.0079 (8)	0.0140 (7)
Cl2	0.0919 (14)	0.0452 (10)	0.0497 (10)	−0.0193 (9)	−0.0122 (9)	0.0256 (8)
Cl3	0.0447 (9)	0.0490 (9)	0.0523 (10)	−0.0104 (7)	−0.0098 (8)	−0.0073 (7)
Cl4	0.0585 (12)	0.0893 (14)	0.0474 (10)	0.0312 (10)	0.0165 (8)	−0.0046 (9)
Cl5	0.0737 (14)	0.0646 (12)	0.0854 (15)	−0.0219 (10)	−0.0323 (11)	0.0147 (10)
Cl6	0.0885 (15)	0.0613 (11)	0.0630 (12)	0.0274 (10)	0.0125 (11)	−0.0232 (9)
N1	0.056 (3)	0.042 (3)	0.030 (3)	0.005 (2)	0.003 (2)	0.001 (2)
N2	0.054 (3)	0.028 (3)	0.049 (3)	−0.003 (2)	0.006 (3)	−0.002 (2)
O1	0.100 (4)	0.038 (3)	0.036 (3)	−0.001 (2)	0.012 (2)	−0.010 (2)
O2	0.086 (4)	0.035 (2)	0.042 (3)	0.006 (2)	0.013 (2)	0.012 (2)
C1	0.051 (4)	0.050 (4)	0.031 (3)	−0.007 (3)	0.005 (3)	0.001 (3)
C2	0.036 (3)	0.045 (4)	0.029 (3)	0.005 (3)	0.002 (3)	0.003 (3)
C3	0.033 (3)	0.032 (3)	0.028 (3)	−0.002 (2)	−0.001 (2)	−0.005 (2)
C4	0.066 (4)	0.021 (3)	0.038 (4)	0.005 (3)	0.005 (3)	−0.006 (2)
C5	0.063 (4)	0.024 (3)	0.036 (3)	0.001 (3)	0.004 (3)	0.000 (2)
C6	0.037 (3)	0.034 (3)	0.025 (3)	0.000 (3)	0.003 (2)	−0.005 (2)
C7	0.048 (4)	0.021 (3)	0.038 (3)	0.003 (2)	0.004 (3)	−0.004 (2)
C8	0.043 (4)	0.033 (3)	0.029 (3)	0.001 (3)	−0.002 (3)	0.001 (2)
C9	0.097 (6)	0.025 (3)	0.061 (5)	0.004 (3)	0.018 (4)	0.006 (3)
C10	0.039 (4)	0.030 (3)	0.040 (4)	0.005 (3)	0.001 (3)	0.004 (3)
C11	0.030 (3)	0.027 (3)	0.030 (3)	−0.001 (2)	0.000 (2)	0.001 (2)
C12	0.042 (3)	0.027 (3)	0.032 (3)	−0.002 (2)	0.006 (3)	−0.004 (2)
C13	0.044 (4)	0.034 (3)	0.031 (3)	−0.006 (3)	0.006 (3)	0.001 (3)
C14	0.030 (3)	0.025 (3)	0.038 (3)	−0.003 (2)	0.002 (3)	0.003 (2)
C15	0.053 (4)	0.031 (3)	0.035 (3)	−0.002 (3)	0.007 (3)	−0.009 (2)
C16	0.049 (4)	0.033 (3)	0.029 (3)	−0.001 (3)	0.006 (3)	0.003 (2)

### Geometric parameters (Å, °)

**Table:**

Sn1—Cl5	2.3873 (18)	C4—C5	1.373 (8)
Sn1—Cl6	2.3938 (17)	C4—H4	0.93
Sn1—Cl4	2.4090 (17)	C5—C6	1.378 (7)
Sn1—Cl1	2.4158 (15)	C5—H5	0.93
Sn1—Cl3	2.4206 (15)	C6—C7	1.360 (7)
Sn1—Cl2	2.4346 (15)	C7—C8	1.379 (8)
N1—C6	1.486 (7)	C7—H7	0.93
N1—H1A	0.89	C8—H8	0.93
N1—H1B	0.89	C9—C10	1.471 (9)
N1—H1C	0.89	C9—H9A	0.96
N2—C14	1.482 (7)	C9—H9B	0.96
N2—H2A	0.89	C9—H9C	0.96
N2—H2B	0.89	C10—C11	1.509 (7)
N2—H2C	0.89	C11—C16	1.381 (8)
O1—C2	1.220 (7)	C11—C12	1.390 (8)
O2—C10	1.217 (7)	C12—C13	1.389 (7)
C1—C2	1.480 (8)	C12—H12	0.93
C1—H1D	0.96	C13—C14	1.365 (8)
C1—H1E	0.96	C13—H13	0.93
C1—H1F	0.96	C14—C15	1.378 (8)
C2—C3	1.502 (8)	C15—C16	1.384 (8)
C3—C4	1.389 (8)	C15—H15	0.93
C3—C8	1.390 (7)	C16—H16	0.93
			
Cl5—Sn1—Cl6	92.32 (8)	C3—C4—H4	119.6
Cl5—Sn1—Cl4	89.17 (8)	C4—C5—C6	118.4 (5)
Cl6—Sn1—Cl4	178.43 (8)	C4—C5—H5	120.8
Cl5—Sn1—Cl1	90.42 (7)	C6—C5—H5	120.8
Cl6—Sn1—Cl1	90.34 (7)	C7—C6—C5	122.5 (5)
Cl4—Sn1—Cl1	89.16 (7)	C7—C6—N1	118.8 (5)
Cl5—Sn1—Cl3	178.91 (8)	C5—C6—N1	118.6 (5)
Cl6—Sn1—Cl3	88.53 (7)	C6—C7—C8	118.7 (5)
Cl4—Sn1—Cl3	89.98 (7)	C6—C7—H7	120.6
Cl1—Sn1—Cl3	90.25 (6)	C8—C7—H7	120.6
Cl5—Sn1—Cl2	90.16 (7)	C7—C8—C3	120.5 (5)
Cl6—Sn1—Cl2	89.33 (7)	C7—C8—H8	119.7
Cl4—Sn1—Cl2	91.16 (7)	C3—C8—H8	119.7
Cl1—Sn1—Cl2	179.35 (7)	C10—C9—H9A	109.5
Cl3—Sn1—Cl2	89.18 (6)	C10—C9—H9B	109.5
C6—N1—H1A	109.5	H9A—C9—H9B	109.5
C6—N1—H1B	109.5	C10—C9—H9C	109.5
H1A—N1—H1B	109.5	H9A—C9—H9C	109.5
C6—N1—H1C	109.5	H9B—C9—H9C	109.5
H1A—N1—H1C	109.5	O2—C10—C9	121.4 (5)
H1B—N1—H1C	109.5	O2—C10—C11	118.9 (5)
C14—N2—H2A	109.5	C9—C10—C11	119.7 (5)
C14—N2—H2B	109.5	C16—C11—C12	119.4 (5)
H2A—N2—H2B	109.5	C16—C11—C10	118.7 (5)
C14—N2—H2C	109.5	C12—C11—C10	121.9 (5)
H2A—N2—H2C	109.5	C13—C12—C11	120.8 (5)
H2B—N2—H2C	109.5	C13—C12—H12	119.6
C2—C1—H1D	109.5	C11—C12—H12	119.6
C2—C1—H1E	109.5	C14—C13—C12	118.1 (5)
H1D—C1—H1E	109.5	C14—C13—H13	121.0
C2—C1—H1F	109.5	C12—C13—H13	121.0
H1D—C1—H1F	109.5	C13—C14—C15	122.8 (5)
H1E—C1—H1F	109.5	C13—C14—N2	119.2 (5)
O1—C2—C1	121.4 (5)	C15—C14—N2	118.0 (5)
O1—C2—C3	120.0 (5)	C14—C15—C16	118.5 (5)
C1—C2—C3	118.6 (5)	C14—C15—H15	120.7
C4—C3—C8	119.0 (5)	C16—C15—H15	120.7
C4—C3—C2	120.3 (5)	C11—C16—C15	120.5 (5)
C8—C3—C2	120.8 (5)	C11—C16—H16	119.8
C5—C4—C3	120.7 (5)	C15—C16—H16	119.8
C5—C4—H4	119.6		

### Hydrogen-bond geometry (Å, °)

**Table:**

*D*—H···*A*	*D*—H	H···*A*	*D*···*A*	*D*—H···*A*
N2—H2A···O1^i^	0.89	2.06	2.939 (7)	170
N1—H1B···O2^ii^	0.89	2.01	2.884 (6)	168
N1—H1A···Cl1^iii^	0.89	2.49	3.322 (6)	156
N2—H2B···Cl2^i^	0.89	2.59	3.350 (6)	144
N2—H2C···Cl3^iv^	0.89	2.69	3.321 (5)	129
N1—H1C···Cl5^v^	0.89	2.55	3.367 (5)	153
N2—H2C···Cl6^iv^	0.89	2.64	3.442 (5)	151

Symmetry codes: (i) −*x*+1, *y*−1/2, −*z*+1/2; (ii) −*x*+1, −*y*+1, −*z*; (iii) *x*+1, −*y*+1/2, *z*−1/2; (iv) −*x*, *y*−1/2, −*z*+1/2; (v) *x*, −*y*+1/2, *z*−1/2.

## References

[bb1] Antonietti, M. & Ozin, G. A. (2004). *Chem. Eur. J.* **10**, 28–41. 10.1002/chem.200305009 14695547

[bb2] Bruker (1997). *SMART* and *SAINT* Bruker AXS Inc., Madison, Wisconsin, USA.

[bb3] Cong, H. P. & Yu, S. H. (2009). *Curr. Opin. Colloid Interface Sci.* **14**, 71–80.

[bb4] Descazo, A. B., Martinez-Manez, R., Sancenón, F., Hoffmann, K. & Rurack, K. (2006). *Angew. Chem. Int. Ed.* **45**, 5924–5948. 10.1002/anie.200600734 16955396

[bb5] Li, Y. Y., Zheng, G. L., Lin, C. K. & Lin, J. (2007). *Solid State Sci.* **9**, 855–861.

[bb6] Sanchez, C., Julián, B., Belleville, P. & Popall, M. (2005). *J. Mater. Chem.* **15**, 3559–3592.

[bb7] Sheldrick, G. M. (1996). *SADABS* University of Göttingen, Germany.

[bb8] Sheldrick, G. M. (2008). *Acta Cryst.* A**64**, 112–122. 10.1107/S0108767307043930 18156677

